# Targeted Quantification of Phosphorylation Sites Identifies STRIPAK-Dependent Phosphorylation of the Hippo Pathway-Related Kinase SmKIN3

**DOI:** 10.1128/mBio.00658-21

**Published:** 2021-05-04

**Authors:** Valentina Stein, Bernhard Blank-Landeshammer, Ramona Märker, Albert Sickmann, Ulrich Kück

**Affiliations:** a Allgemeine und Molekulare Botanik, Ruhr-Universität, Bochum, Germany; b Leibniz-Institut für Analytische Wissenschaften-ISAS-e.V., Dortmund, Germany; University of Melbourne

**Keywords:** phosphorylation site occupancy, striatin-interacting phosphatase and kinase (STRIPAK) complex, phosphoproteome, *Sordaria macrospora*, protein phosphatase 2 (PP2A), protein phosphorylation, serine/threonine protein kinase, fungi, cell differentiation

## Abstract

We showed recently that the germinal center kinase III (GCKIII) SmKIN3 from the fungus Sordaria macrospora is involved in sexual development and hyphal septation. Our recent extensive global proteome and phosphoproteome analysis revealed that SmKIN3 is a target of the striatin-interacting phosphatase and kinase (STRIPAK) multisubunit complex. Here, using protein samples from the wild type and three STRIPAK mutants, we applied absolute quantification by parallel-reaction monitoring (PRM) to analyze phosphorylation site occupancy in SmKIN3 and other septation initiation network (SIN) components, such as CDC7 and DBF2, as well as BUD4, acting downstream of SIN. For SmKIN3, we show that phosphorylation of S668 and S686 is decreased in mutants lacking distinct subunits of STRIPAK, while a third phosphorylation site, S589, was not affected. We constructed SmKIN3 mutants carrying phospho-mimetic and phospho-deficient codons for phosphorylation sites S589, S668, and S686. Investigation of hyphae in a Δ*Smkin3* strain complemented by the S668 and S686 mutants showed a hyper-septation phenotype, which was absent in the wild type, the Δ*Smkin3* strain complemented with the wild-type gene, and the S589 mutant. Furthermore, localization studies with SmKIN3 phosphorylation variants and STRIPAK mutants showed that SmKIN3 preferentially localizes at the terminal septa, which is distinctly different from the localization of the wild-type strains. We conclude that STRIPAK-dependent phosphorylation of SmKIN3 has an impact on controlled septum formation and on the time-dependent localization of SmKIN3 on septa at the hyphal tip. Thus, STRIPAK seems to regulate SmKIN3, as well as DBF2 and BUD4 phosphorylation, affecting septum formation.

## INTRODUCTION

The striatin-interacting phosphatase and kinase (STRIPAK) multisubunit complex functions as a macromolecular assembly communicating through physical interactions with other conserved signaling protein complexes to constitute larger dynamic protein networks. STRIPAK is involved in a broad variety of developmental processes in higher and lower eukaryotes. For example, proliferation of several mammalian cancer cells is correlated with dysfunctional STRIPAK subunits (1–3), and in fungal microorganisms, the lack of STRIPAK results in sexual infertility, defects in hyphal fusion, reduced secondary metabolite production, and impaired pathogenicity or symbiotic interactions (4, 5).

We are interested in identifying putative phosphorylation and dephosphorylation targets of STRIPAK in the filamentous fungus Sordaria macrospora, a filamentous ascomycete closely related to Neurospora crassa (6). Techniques to globally quantify the proteome and phosphoproteome, such as label-free quantification (LFQ) and label-based approaches (isobaric tags for relative and absolute quantitation [iTRAQ] and tandem mass tags [TMTs]), are indispensable for large-scale detection of changes in the phosphorylation of peptides and the identification of potential molecular targets of kinases and phosphatases (7). We recently performed extensive isobaric tagging for relative- and absolute-quantification-based proteomic and phosphoproteomic analyses to identify potential targets of STRIPAK in *S. macrospora*: the proteome and phosphoproteome of the wild type and three different STRIPAK deletion mutants lacking either the striatin homolog PRO11, the striatin-interacting protein PRO22, or the catalytic PP2A subunit PP2Ac1. This proteomic analysis revealed a total of 4,193 proteins and 2,489 phosphoproteins in all strains; 1,727 proteins were present in both proteomes. Among these, we identified 781 phosphoproteins in all mutants that showed phosphorylation different from that in the wild type (8). However, the functional role of the posttranslational protein phosphorylation was characterized in only a few cases (8, 9).

Certain inherent limitations with these shotgun methods, such as ratio compression and undersampling, can be overcome only by the complementary use of targeted mass spectrometry (MS) approaches. These can be employed as a means to validate a subset of the results obtained by shotgun experiments. While targeted MS is widely used for accurate protein quantification, the high variability, increased experimental effort, and need for validation has limited the implementation of targeted approaches in phosphoproteomics analysis (10). A hallmark study showed that 25% of differentially regulated phosphopeptides were attributed to alterations at the protein level (11). Thus, to accurately determine the phosphorylation ratio of a given site, targeted quantification of phosphorylation sites is highly appropriate to quantify both the corresponding phosphorylated and nonphosphorylated peptides in order to obtain site-specific phosphorylation ratios (12, 13).

Among the 781 regulated proteins in *S. macrospora* mentioned above (8), we found the germinal center kinase III (GCKIII) SmKIN3, which, however, was absent in the global proteome and thus did not permit quantitative measurement of phosphorylation. Therefore, SmKIN3 phosphorylation was determined by applying absolute quantification by synthesis of stable isotope-labeled standard (SIS) peptides combined with parallel-reaction monitoring (PRM), a method which has not yet been applied to a fungal organism to identify phosphorylation sites.

SmKIN3 is involved in septation of hyphae and associated with the highly conserved septation initiation network (SIN) complex (14). The SIN complex, homologous to Hippo signaling in animals, comprises a sterile protein (STE) kinase, a GCK, and a nuclear DBF2-related (NDR) kinase (15). For example, in N. crassa, the STE kinase CDC-7 phosphorylates GCK SID-1, the homolog of SmKIN3, and activates DBF-2 (16). The function of SIN is essential for septation and cytokinesis, as demonstrated by SIN deletion strains (14, 16, 17). Bud4, an anillin-related protein, acts further downstream and specifies SIN-regulated septum formation (18, 19).

Here, we show that STRIPAK-directed phosphorylation of SmKIN3 has a significant impact on proper hyphal septation and septal localization. This is the first report about STRIPAK-dependent phosphorylation analyzed by targeted quantification of phosphorylation sites, and it will have an impact on understanding the function of mammalian homologs.

## RESULTS

### Absolute quantification of phosphorylation site occupancy by PRM of germinal center kinase SmKIN3.

**FIG 1:**
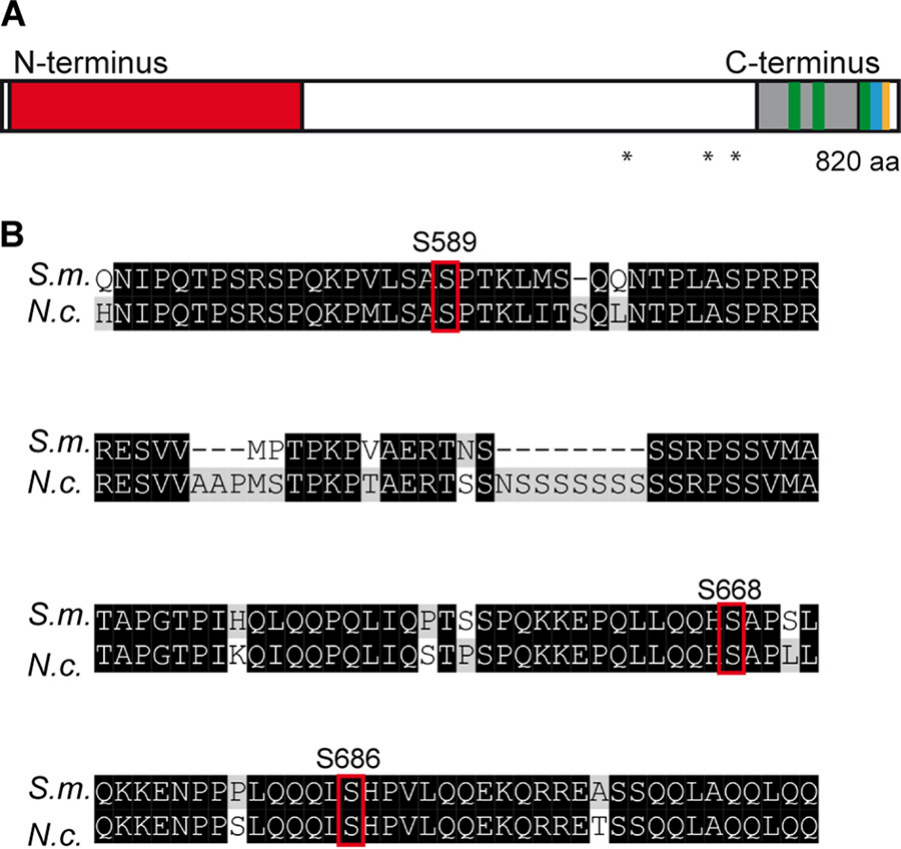
(A) Linear structure of SmKIN3 and identified protein domains. Depicted are a serine/threonine kinase domain (aa 10 to 279, red), large tumor suppressor kinase 1 (LATS1) kinase phosphorylation motifs (aa 721 to 727, 740 to 746, and 794 to 800; green), a coiled-coil domain (aa 688 to 788; gray), a T-motif (aa 801 to 806; blue), and a phospho-threonine motif, binding a subset of FHA domains (aa 805 to 811; yellow). Asterisks below indicate phosphorylation sites S589, S668, and S686. (B) Alignment of sequences of SmKIN3 from *S. macrospora* (*S.m.*) and SID-1 from Neurospora crassa (*N.c*.). Phosphorylation sites were framed, which were investigated by absolute quantification of protein phosphorylation.

Previously, we showed that the GCKIII SmKIN3 is associated with STRIPAK and regulates fungal development (14). Sequence comparison of the primary amino acid sequence showed a homology of 92.43% with the corresponding sequence from N. crassa, but similarity with other homologues from ascomycetes is low. Using the eukaryotic linear motif (ELM) database (20), we found a kinase domain in the amino terminus, several large tumor suppressor (LATS) kinase recognition motifs, and a binding motif for forkhead-associated (FHA) domains. The COILS sequence analysis program (http://www.ch.embnet.org/software/COILS_form.html) revealed two predicted putative coiled-coil domains located next to each other in a region between amino acids (aa) 688 and 788. At the C-terminal end of SmKIN3 (aa 805 to 811), we detected a conserved sequence motif, previously called the T-motif (Fig. 1A). Such T-motifs have so far been found only in a small family of related fungal kinases (21). Database research revealed that this motif occurs, for example, in SmKIN3, its homologue Sid1p from Schizosaccharomyces pombe, and SID-1 from N. crassa.

Recently, we determined the phosphoproteomes of wild-type and STRIPAK mutant S. macrospora strains (8) and detected three phosphorylation sites, S589, S668, and S686, in the SmKIN3 sequence, which are conserved between *S. macrospora* and N. crassa (Fig. 1B). Due to its low abundance in the overall proteome, we were unable to quantify the STRIPAK-dependent phosphorylation of SmKIN3 (8).

Here, we set out to determine the impact of STRIPAK on SmKIN3 phosphorylation by applying targeted quantification of phosphorylation site occupancy using PRM. As described above, SmKIN3, together with CDC7 and DBF2, constitutes the SIN complex, which is associated with the downstream landmark protein BUD4. Therefore, we included all these components in our PRM analysis.

A targeted bipartite TiO_2_-liquid chromatography (LC)-PRM-based workflow was established to quantify the site-specific phosphorylation states of putative STRIPAK targets. First, 70 phosphorylated peptides and their corresponding nonphosphorylated counterparts were selected, and SIS were synthesized. The median coefficients of variation (CV) of the biological replicates were calculated as 16.3% for all nonphosphorylated peptides and 16.1% for all phosphorylated peptides. To further prioritize our approach, we selected 15 pairs of peptides, representing phospho sites from proteins belonging to the SIN signaling pathway. Dilution series were prepared to verify a linear response and determine the limit of detection (LOD) of each SIS peptide, as described in Materials and Methods. LODs ranged from 1.7 amol to 19.3 fmol per injection for phosphorylated SIS peptides and from 1.43 amol to 32.3 fmol per injection for their nonphosphorylated counterparts. The average lower limit of quantification (LLOQ) was 375 amol or 135 amol on column for phosphorylated or nonphosphorylated peptides. The median CV throughout all dilution steps was calculated as 6.7% for phosphorylated peptides and 5.0% for nonphosphorylated peptides. These results are summarized in Data Set S1 in the supplemental material.

10.1128/mBio.00658-21.8 DATA SET S1 Data from the PRM analysis. Download Data Set S1, XLSX file, 0.3 MB. Copyright © 2021 Stein et al. 2021 Stein et al. https://creativecommons.org/licenses/by/4.0/ This content is distributed under the terms of the Creative Commons Attribution 4.0 International license.

**FIG 2:**
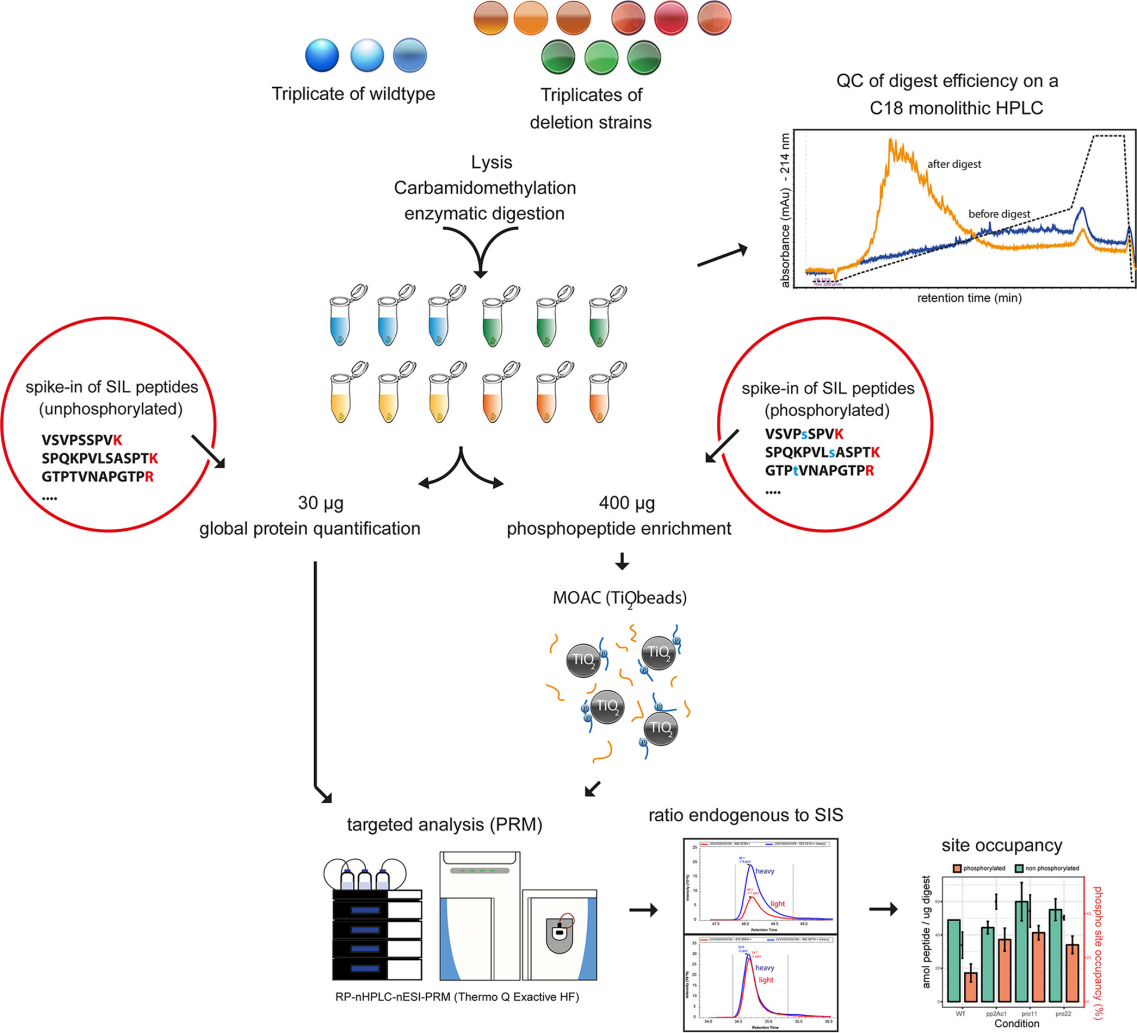
Targeted quantification of phosphorylation site occupancy. Graphical representation of the proteomics workflow, including (i) extraction and digestion of proteins from *S. macrospora* strains grown in triplicates, (ii) spike-in of phosphorylated and unphosphorylated SIS peptides, (iii) TiO_2_-based enrichment of phosphorylated peptides, (iv) targeted analysis using a reversed-phase nano-HPLC–nano-electrospray ionization–PRM (RP-nHPLC-nESI-PRM) setup, and (v) data analysis and calculation of phosphorylation site occupancy. QC, quality control; MOAC, metal oxide affinity chromatography.

The *S. macrospora* wild type and the three STRIPAK deletion strains, the Δ*pro11*, Δ*pro22*, and Δ*pp2Ac1* mutants, were grown in triplicates prior to being subjected to lysis and protein extraction (Fig. 2). After tryptic digestion and quality control measurements, protein amounts were normalized and aliquots were spiked with either phosphorylated SIS peptides or their nonphosphorylated counterparts. The phosphopeptide aliquots were subjected to TiO_2_-based enrichment followed by LC-PRM measurement, while the fraction spiked with the nonphosphorylated peptides was measured directly. The targeted PRM measurements of SIS and endogenous peptides allowed the simultaneous quantification of both the phosphorylated and the nonphosphorylated peptide counterparts and thus enabled us to calculate the phosphorylation site occupancy.

Using the proteomics workflow described above, we were able to distinguish three different outcomes.

First, we determined the site occupancy for 37 phosphorylation sites on 4 proteins, where the phosphorylated and nonphosphorylated peptides were determined. Included is phosphorylation site S686 from SmKIN3. For DBF2, we detected four phosphorylation sites, three of which were quantified. Phosphorylation site S104 in DBF2 was significantly decreased when STRIPAK was nonfunctional, indicating that this site is STRIPAK dependent. Phosphorylation of sites S89 and S502 was not differentially regulated in STRIPAK deletion mutants (Data Set S2). We found five phosphorylation sites in BUD4 (T381, S367, S369, S742, S1373); all were dephosphorylated in STRIPAK deletion mutants (Data Set S2).

10.1128/mBio.00658-21.9 DATA SET S2 Data from phosphosite quantification. Download Data Set S2, XLSX file, 1.1 MB. Copyright © 2021 Stein et al. 2021 Stein et al. https://creativecommons.org/licenses/by/4.0/ This content is distributed under the terms of the Creative Commons Attribution 4.0 International license.

Second, nine phosphorylation sites were detected on five proteins, but the nonphosphorylated peptide was not detectable. However, the phosphorylation sites were quantified by detection of the phosphorylated peptide and at least one corresponding pair of phosphorylated and nonphosphorylated peptides from the same protein. The phosphorylation site occupancy for these peptides was indirectly calculated based on the concentration of the remaining peptide pairs of the respective protein. Among these, we found two sites (S668, S589) in the SmKIN3 protein.

Finally, as a third option, we found seven phosphorylation sites on five proteins, where only the phosphorylated peptides were detected and no site occupancy value could be calculated (Data Set S2). However, changes in the abundances of selected phosphorylated peptides could still be used for quantification, since the corresponding proteins were stably expressed in the wild type and STRIPAK deletion mutants in previous experiments. An example is the CDC7 protein, which was recently detected in a phosphoproteomic analysis (8).

**FIG 3:**
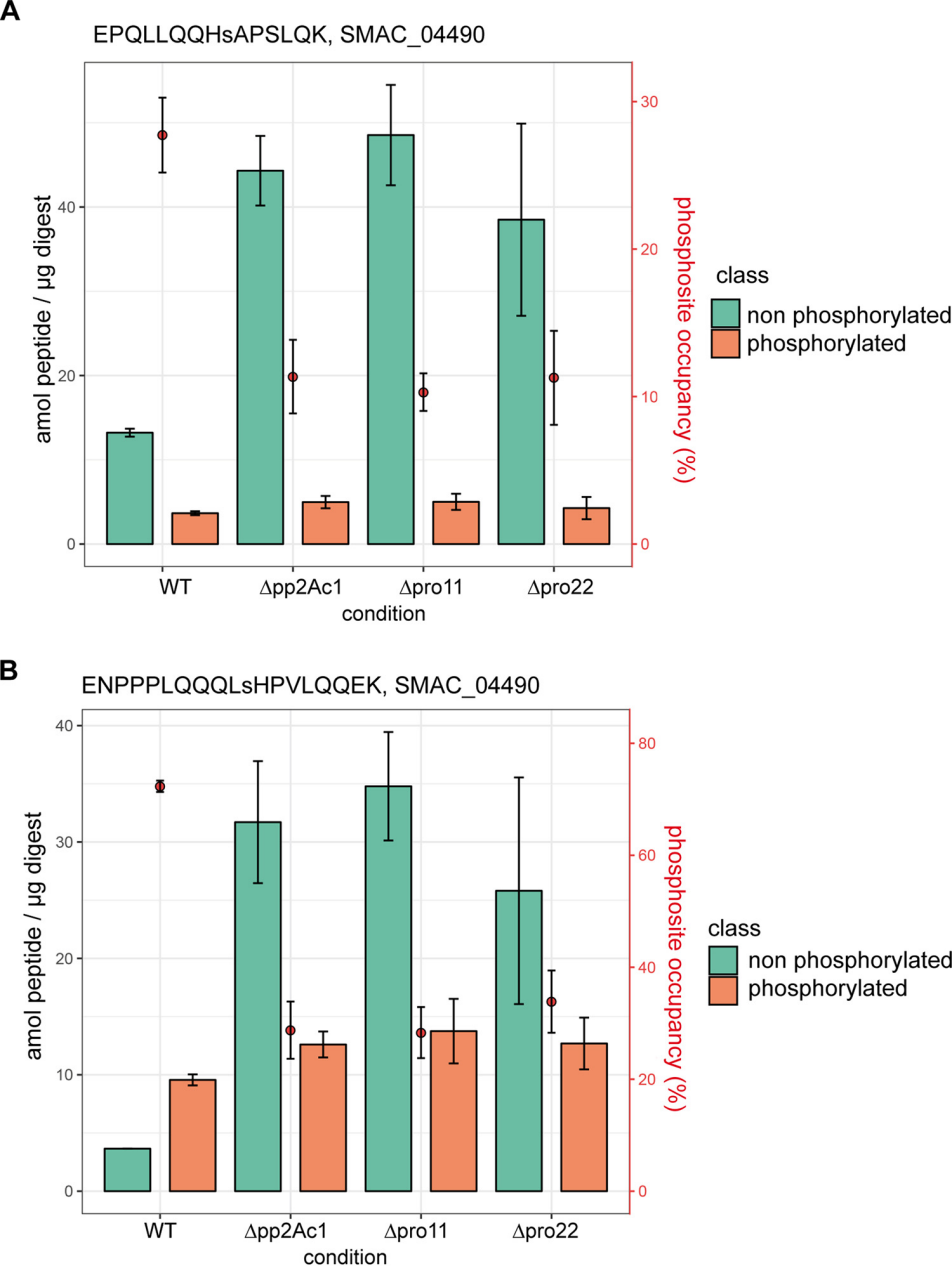
(A, B) Quantitative analysis of the nonphosphorylated and phosphorylated peptides containing phosphorylation sites S668 and S686 of SmKIN3 in three different STIPAK deletion mutants. The *y* axis on the left gives the amounts of the peptides EPQLLQQHsAPSLQK and ENPPPLQQQLHsHPVLQQEK in attomoles as nonphosphorylated (green) and phosphorylated variants (orange). The *y* axis on the right shows the quantity of phosphosite occupancy as a percentage, plotted as red dots in the bar chart. Error bars indicate standard deviations. Lowercase letters in the peptides indicate the phosphosite.

Overall, 19 phosphorylation sites showed significantly different site occupancy between the wild type and at least one of the STRPIAK mutant strains (Student's *t*-test *P* value < 0.05; the results are summarized in Data Set S2). This quantification of site occupancy helped us to prioritize our functional analyses of phosphorylation sites from SmKIN3, the main objective of this investigation. It was also the source for constructing a mechanistic model of phosphorylation-dependent protein regulation. The number of peptides where both phosphorylation sites S668 and S686 are dephosphorylated in STRIPAK deletion mutants is higher than in the wild type; i.e., peptides containing phosphorylated sites S668 and S686 are less abundant in STRIPAK deletion mutants than in the wild type (Fig. 3A and B). In contrast, we detected only a slightly higher number of peptides with phosphorylation at site S589, indicating that this site is not STRIPAK dependent (Fig. S1).

10.1128/mBio.00658-21.4 FIG S1 Quantitative analysis of the nonphosphorylated and phosphorylated peptides containing phosphorylation site S589 of SmKIN3 in three different STRIPAK deletion mutants. The *y* axis on the left side gives the amount of the peptide SPDKPVLSAsPTK in attomoles as a nonphosphorylated variant (green) and phosphorylated variant (orange). The *y* axis on the right side shows the quantity of phosphosite occupancy in percentages as red dots. Lowercase letter in the peptide indicates the phosphosite. Error bars indicate standard deviations. Download FIG S1, TIF file, 0.6 MB. Copyright © 2021 Stein et al. 2021 Stein et al. https://creativecommons.org/licenses/by/4.0/ This content is distributed under the terms of the Creative Commons Attribution 4.0 International license.

### Phospho-mimetic and phospho-deficient SmKIN3 mutants display a hyper-septation phenotype.

Our quantitative analysis of phosphosite occupancy in SmKIN3 indicated that SmKIN3 is a target of STRIPAK. To analyze the function of phosphorylated SmKIN3, we generated six different phospho-deficient plus phospho-mimetic mutants by subjecting the triplets encoding S589, S668, and S686 to *in vitro* mutagenesis, replacing the serine triplets with either alanine (no phosphorylation) or glutamic acid (mimics phosphorylation) triplets, as described in Materials and Methods. The phosphorylation of S589 is apparently STRIPAK independent (Fig. S1), while S668 and S686 seem to depend on STRIPAK (Fig. 3).

**FIG 4:**
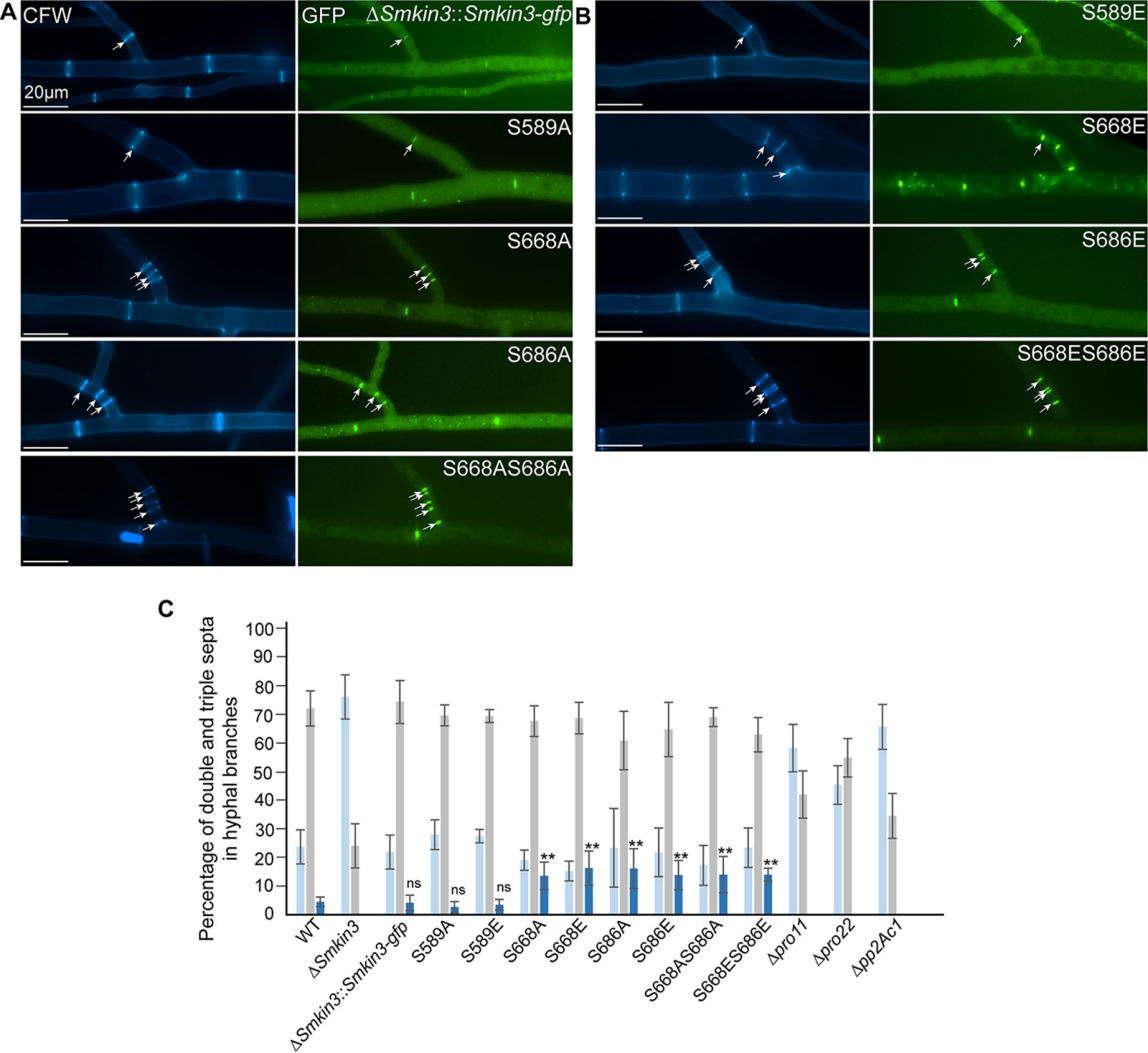
Hyper-septation phenotype in the wild type (WT) and phospho-mutants. (A, B) Fluorescence microscopic investigation of septation in hyphal branches of strains, as indicated. Mycelia were stained with CFW, and SmKIN3 is labeled with GFP. (C) Quantitative investigation of hyper-septation. All values are given as percentages. We investigated at least 100 hyphal branches for each STRIAPK deletion strain. In the case of recombinant strains, three different single-ascospore isolates were investigated to exclude side effects from random integration of recombinant DNA into genomic DNA. In these cases, at least 200 hyphal branches were counted for each recombinant strain. As a control, 600 hyphae from the Δ*Smkin3* strain complemented with the wild-type gene and Δ*Smkin3* strains were investigated in three technical replicates. Percentages of hyper-septation (2 to 5 septa) are given in dark blue; one septum is indicated in grey, and no septa is indicated in light blue. Error bars indicate standard deviations. Significant differences of hyper-septation from that of the wild type are indicated by asterisks and were evaluated by a two-sided Student *t*-test (**, *P ≥ *0.01; ns, not significant).

For functional analysis of SmKIN3 and its variants, we used a previously described *Smkin3* deletion strain for complementation analysis (14). Recombinant plasmids (Table S1) encoding SmKIN3-GFP phospho-variants were transformed into the *ΔSmkin3* deletion strain, and primary transformants were used to isolate homokaryotic ascospores. The expression of wild-type SmKIN3-GFP and phospho-variants was verified by Western blot analysis, indicating the biosynthesis of a 118-kDa protein (Fig. S2A). When the wild-type, tagged *Smkin3* gene was used for complementation, we obtained fully fertile strains with wild-type mycelial growth, indicating that green fluorescent protein (GFP)-labeled SmKIN3 is fully functional. We then investigated three homokaryotic ascospore isolates of each phospho-mimetic strain, i.e., those with S589E, S668E, and S686E, and phospho-deficient strains, i.e., those with S589A, S668A, and S686A. Growth rates of mutant strains, grown at different temperatures and under different light conditions, resemble that of the wild type (Fig. S3A). Similarly, fluorescence microscopy showed that phospho-mutation SmKIN3 still localizes to septa and close to nuclei; its situation thus resembles that of the wild type (Fig. S3B). However, we found that both the S668A and S668E mutants showed an increased number of septa in hyphal branches, as detected by GFP fluorescence of the SmKIN3-phospho variants. The identities of septa were further confirmed by calcofluor white (CFW) staining. We investigated at least 200 single hyphal branches for each strain in three technical triplicates. In these cases, at least 600 hyphal branches were counted for each recombinant strain (Fig. 4A to C; Data Set S3). We considered all septa within 20 μm of hyphal branches. Double, triple, quadruple, and quintuple septa were counted when they were located within a maximum distance of 12 μm. These are referred to as the hyper-septation phenotype.

10.1128/mBio.00658-21.1 TABLE S1 Plasmids used in this work. Download Table S1, DOCX file, 0.01 MB. Copyright © 2021 Stein et al. 2021 Stein et al. https://creativecommons.org/licenses/by/4.0/ This content is distributed under the terms of the Creative Commons Attribution 4.0 International license.

10.1128/mBio.00658-21.5 FIG S2 (A) Expression control of phospho-mutated variants of SmKIN3 tagged with GFP. Strains were grown for 3 days in liquid medium (malt-cornmeal medium [BMM]) as a surface culture. For each strain, 10 μg of crude protein extract was subjected to SDS-PAGE. Western blot analysis was performed with an anti-GFP antibody and an anti-α-tubulin antibody as control. SmKIN3 tagged with GFP has a mass of 118 kDa, while α-tubulin has a mass of 55 kDa. SmKIN3-GFP was detected in all eight different phospho-mutants (S589A, S589E, S668A, S668E, S686A, S686E, S668A S686A, and S668E S686E mutants). The wild type and a complemented Δ*Smkin3* strain were used as controls and are shown only once, indicated by the black lines. (B) Southern blot analysis of the *Smkin3* copy number in SmKIN3-phospho variants. Genomic DNAs of the wild type, Δ*Smkin3*::*Smkin3-gfp* strain, and S589A, S589E, S668A, S668E, S686A, S686E, S668A S686A, and S668E S686E phospho-mutants were digested with the restriction enzymes *Apa*I and *Pst*I for hybridization with *Smkin3.* Autoradiograph of Southern blot hybridization with radioactively labeled probe specific for *Smkin3.* The wild-type *Smkin3* band is visible at 7241 bp and the recombinant *Smkin3* gene is visible at 5757 bp. The wild type and a complemented Δ*Smkin3* strain were used as controls and are shown only once, indicated by the black lines. Download FIG S2, TIF file, 1.2 MB. Copyright © 2021 Stein et al. 2021 Stein et al. https://creativecommons.org/licenses/by/4.0/ This content is distributed under the terms of the Creative Commons Attribution 4.0 International license.

10.1128/mBio.00658-21.6 FIG S3 Characterization of the wild type and mutant strains. (A) Phenotypic characterization of the growth rate and fertility of strains, as indicated, when grown at 27°C under light conditions. The phospho-mutants resemble the wild type when grown under different physiological conditions, as noted in Materials and Methods. (B) The SmKIN3-S686E protein localizes to septa of vegetative hyphae (white arrows) having different ages and close to the nucleus at spindle pole bodies (red arrows). In addition, SmKIN3 appears at septa of vegetative hyphae and localizes as well to septa of young and mature hyphae marked by a white arrow. Strains were grown for 1 day on a solid-BMM-coated glass. Samples were stained with calcofluor white M2R (Sigma-Aldrich; 1 μg/μl diluted 1:400). The image serves as an example for all phospho-mutated strains, as well as for the complemented *Smkin3* strain and both the Δ*pro11*::*Smkin3-gfp* and Δ*pp2Ac1*::*Smkin3-gfp* strains. Download FIG S3, PDF file, 0.5 MB. Copyright © 2021 Stein et al. 2021 Stein et al. https://creativecommons.org/licenses/by/4.0/ This content is distributed under the terms of the Creative Commons Attribution 4.0 International license.

10.1128/mBio.00658-21.10 DATA SET S3 Data for quantification of the hyper-septation phenotype. Download Data Set S3, XLSX file, 0.01 MB. Copyright © 2021 Stein et al. 2021 Stein et al. https://creativecommons.org/licenses/by/4.0/ This content is distributed under the terms of the Creative Commons Attribution 4.0 International license.

In the case of hyper-septation, the value for the *S. macrospora* wild type was 4.4% ± 1.6%. The hyper-septation value for the complemented strains and phospho-mutants were as follows: for the Δ*Smkin3*::*Smkin3-gfp* strain, 4.0% ± 2.7%; for the S589A phospho-mutant, 2.6% ± 1.9%; for the S589E phospho-mutant, 3.4% ± 1.9%; for the S668A phospho-mutant, 13.5% ± 4.8%; for the S668E phospho-mutant, 16.2% ± 6.0%; for the S686A phospho-mutant, 16.0% ± 7.0%; and for the S686E phospho-mutant, 13.7% ± 5.1%. The values (dark blue in Fig. 4C) for the S668 and S686 phospho-mutants significantly deviate from those of the wild type and S589 phospho-mutants.

To verify that both S668 and S686 mutants act similarly on the septation process, we constructed phospho-mimetic (S668E S686E) and phospho-deficient (S668A S686A) double mutants and analyzed derived ascospore isolates. The strains were fully fertile, and the phospho-mutated SmKIN3 proteins are still found at septa and at nuclei in young hyphae (Fig. S3). We found that the corresponding values for hyper-septations of the double mutants (S668A S686A mutant, 13.9% ± 6.4%; S668E S686E mutant, 14.0% ± 2.3%) deviate not significantly from those of single mutants.

From our quantitative results, we conclude that the phospho-mimetic and phospho-deficient SmKIN3 mutations at S686 and S668 are responsible for the hyper-septation phenotype, while the S589 phosphorylation site has no effect on septum formation. To analyze the impact of STRIPAK on septum formation, we examined three STRIPAK deletion strains lacking either the regulatory subunit of the STRIPAK phosphatase (Δ*pro11* strain), the catalytic subunit of the STRIPAK phosphatase (Δ*pp2Ac1* strain), or the STRIP1/2 homologue (Δ*pro22* strain). We found no hyper-septation in the Δ*pro11*, Δ*pro22*, or Δ*pp2Ac1* strain (Fig. 4C). We conclude that septum formation is reduced in STRIPAK deletion mutants and thus is probably positively regulated by STRIPAK.

### Localization of SmKIN3 at septa is dependent on phosphorylation and on an intact STRIPAK complex.

**FIG 5:**
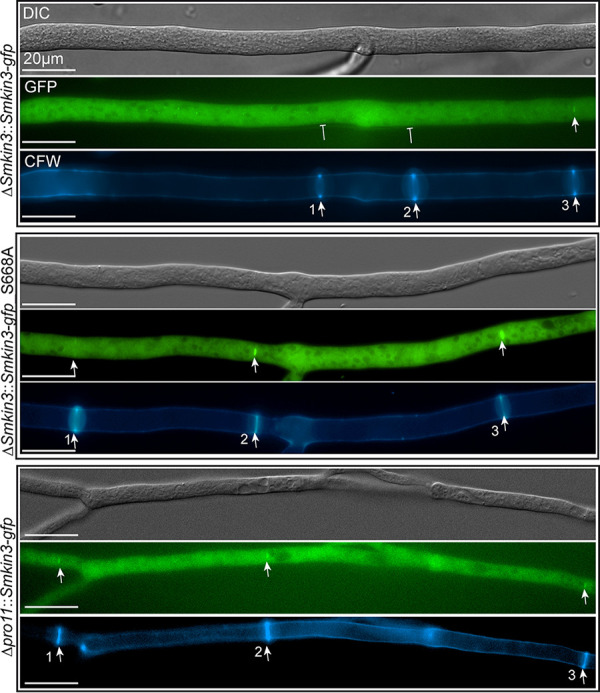
STRIPAK-dependent localization of SmKIN3-GFP at hyphal septa. The strains shown here are indicated on the left. The first three septa from the hyphal tip (to the left) are numbered in the CFW pictures. Arrowheads label green fluorescing SmKIN3 (GFP) or CFW-stained septa. SmKIN3-GFP localization was considered only when it was detected with an exposure time between 100 and 1,000 ms. Inhibiting arrows point to septa where SmKIN3 is missing. DIC, differential interference contrast.

Next, we investigated the effect of the phosphorylation of SmKIN3 on its cellular localization at septa. For fluorescence and differential interference microscopy, we used all eight phosphorylation variants as well as the wild type and two deletion strains lacking genes for STRIPAK subunits (Δ*pro11* and Δ*pp2Ac1* deletion strains). We selected the four terminal septa at the hyphal tip for our detailed localization analysis. We counted the first terminal septa at hyphal tips, where GFP fluorescence indicated SmKIN3 localization (representative images in Fig. 5). As an example, more than 50% of wild-type septa showed fluorescence at the 3rd terminal septum (Table 1). Similar values were obtained for the Δ*Smkin3* strain complemented with the wild-type *Smkin3* gene. Δ*Smkin3* strains carrying the mutated codon S589 were similar; most of the localization was also observed at the 2nd and 3rd septa. A significantly different result was observed in strains with changes to amino acid S668 or S686 as well as the double S668 and S686 combination. Here, we found preferential localization at the 1st or 2nd septum (Fig. S4).

**TABLE 1:** Localization of SmKIN3 and its phosphorylation variants in the wild type and STRIPAK deletion strains

Strain	% of SmKIN3 at the^*a*^:			
	1st septum	2nd septum	3rd septum	4th septum
Wild type::*Smkin3-gfp* strain^*b*^	–	35.1	51.5	13.4
Δ*Smkin3*::*Smkin3-gfp* strain	–	22.0	64.0	14.0
S589A mutant	4.0	47.5	46.5	2.0
S589E mutant	–	43.8	46.6	9.6
S668A mutant	53.5	46.5	–	–
S668E mutant	27.4	67.8	4.8	–
S686A mutant	52.3	47.4	–	–
S686E mutant	44.8	47.8	7.4	–
S668A S686A mutant	26.8	64.3	8.9	–
S668E S686E mutant	39.3	53.6	7.1	–
Δ*pro11*::*Smkin3-gfp* strain^*b*^	68.0	32.0	–	–
Δ*pp2Ac1*::*Smkin3-gfp* strain^*b*^	84.0	16.0	–	–

a In the wild type, SmKIN3 is preferentially seen at the third terminal septum, while in the three variants (the S668, S686, and S668 S686 strains) and both STRIPAK mutants (the Δ*pp2Ac1* and Δ*pro11* strains), localization is observed mostly at the first or second septum. *n* ≥ 50 per strain. Localization of SmKIN3 in the complemented Δ*Smkin3* strain and in both S589 phospho-variants resembles that in the wild type. Septa were stained with CFW, and SmKIN3-GFP localization was considered only when it was detected with an exposure time between 100 and 1,000 ms. –, measured but not detected.

b The strains are isogenic.

10.1128/mBio.00658-21.7 FIG S4 STRIPAK-dependent localization of SmKIN3-GFP in hyphal septa in the S589A, S589E, and S668E SmKIN3-phospho mutants. The first three septa are shown and marked by numbers 1 to 3. Arrowheads label green fluorescing SmKIN3 (GFP) or CFW-stained septa. Inhibiting arrows point to septa where SmKIN3 is missing. This figure corresponds to Fig. 5 and Table 1 and serves as an example for the S668A S686A and S686A S686E double mutants. Download FIG S4, TIF file, 2.6 MB. Copyright © 2021 Stein et al. 2021 Stein et al. https://creativecommons.org/licenses/by/4.0/ This content is distributed under the terms of the Creative Commons Attribution 4.0 International license.

To further investigate the role of STRIPAK, we analyzed the localization of SmKIN3 in two STRIPAK deletion mutants, the Δ*pro11* and Δ*pp2Ac1* strains. In both deletion strains, SmKIN3 localized mainly at the first or second septum, as with the S668 and S686 phospho-variants and unlike in the wild-type strain. This suggests that temporally controlled phosphorylation at sites S668 and S686 is required for the localization of SmKIN3 to early septation sites (Fig. S4).

## DISCUSSION

Here, we provide analytical molecular evidence that the phosphorylation of SmKIN3 at distinct sites is STRIPAK dependent. Tightly regulated phosphorylation of SmKIN3 is important for its function. We discovered not only that deregulation of SmKIN3 phosphorylation leads to a hyper-septation phenotype but also that temporal phosphorylation of SmKIN3 regulates its septal localization.

We accurately quantified 53 phosphorylated peptides and for 37 of those directly determined the site occupancy of the phosphorylation sites by quantifying the corresponding nonphosphorylated peptide. To date, accurate targeted quantification and determination of phosphorylation site occupancy has been performed in several studies, but these are mostly limited to just a few sites (12, 22–25) or used only crude SIS peptides (26). Since our targeted phosphorylated peptides were found at concentrations lower than 30 amol per μg of protein lysate, this result confirms that we used a very sensitive assay to determine phosphorylation site occupancy in a nonhuman model organism (22).

### STRIPAK regulates SmKIN3 phosphorylation indirectly.

In mammalian cells, Hippo comprises two kinases, GCK MST1/2 and NDR kinase LATS1/2 (27, 28). Recently, it was shown that STRIPAK integrates upstream signals to control the activity of the SmKIN3 homolog MST1/2 for initiating Hippo signaling. Deletion of STRIP1/2, a homolog of *S. macrospora* PRO22, results in upregulation of MST1/2, which led to the conclusion that STRIPAK regulates MST1/2 (27, 29, 30).

In fungi, the Hippo homologous kinase cascade SIN comprises three kinases. For example, in N. crassa, the Ste20 kinase CDC-7 acts upstream of GCK SID-1 and NDR kinase DBF-2 (31). SID-1, the middle component of SIN, is the homolog of SmKIN3 and MST1/2. Our PRM analysis revealed that the phosphorylation state of two amino acid residues of SmKIN3 in STRIPAK deletion mutants differ from the wild-type residues; i.e., phosphorylation in two out of three sites in SmKIN3 is decreased in STRIPAK deletion mutants. This finding is intriguing because STRIPAK is a dephosphorylating complex and because the catalytic subunit PP2Ac1 and also PRO22 and PRO11 are essential for phosphatase activity, since the lack of a single subunit prevents STRIPAK assembly and thus results in a deficiency of phosphatase activity. For example, this was shown for two N. crassa mutants lacking the PRO11 or PRO22 homologue (32).

Additionally, comparable results with fission yeast showed that SIN is negatively regulated by the SIN inhibitory PP2A (SIP) complex, the STRIPAK homolog (33). SIP dephosphorylates the upstream Ste20 kinase Cdc7p, which leads to assembly of SIN. Cdc7p itself phosphorylates Sid1p, the homolog of SmKIN3. Dysfunction of SIP prevents the assembly of SIN and thus abolishes the phosphorylation of Sid1p (33). Sid1p, SID-1, and SmKIN3 resemble each other not only in their posttranslational modifications but also in their functions. As with Sid1p in Schizosaccharomyces pombe, SmKIN3 in *S. macrospora* and SID-1 in N. crassa are required for proper septum formation (16, 33).

### The SIN component SmKIN3 is a positive regulator of septum formation.

In filamentous ascomycetes, such as *S. macrospora*, hyphae are compartmentalized by the formation of septa, which are assembled at an actomyosin-based cortical ring (CR), followed by CR constriction. The CR protein complex includes structural proteins, molecular motors, and signaling enzymes (18). Since SmKIN3 localizes at the center of the hyphal septum around the pore, SmKIN3 may be involved in the function of CR signaling enzymes to activate or inhibit targets via posttranslational modifications, such as phosphorylation. In particular, the presence of SmKIN3 at mature septa suggests additional functions besides septum formation, like transmitting signals, as was hypothesized for BUD4 (34).

Our PRM approach provides evidence that SmKIN3 is dependent on STRIPAK for dephosphorylation. The analysis of phospho-mimetic and phospho-deficient SmKIN3 (S668, S686) strains revealed a hyper-septation phenotype, which was also observed in both double phospho-mutants. In contrast, the S589 phospho-mutants showed a septation phenotype comparable to that of the wild type. Since phosphorylation at distinct SmKIN3 sites is regulated by STRIPAK, we hypothesize that the fine spatiotemporal tuning of the phosphorylation status of S668 and S686 mutants is important for the septation process. Thus, the coordinated formation of septa is impaired in both phospho-mimetic and phospho-deficient mutants. The outcome of our mutant analysis is reminiscent of a comparable analysis in yeast (35), where two phospho-mimetic and phospho-deficient serine mutants showed the same growth phenotype, suggesting the essential function of serine for protein activity. In contrast, two other phospho-mutations displayed a wild-type phenotype, indicating that these sites are not critical or regulated by phosphorylation.

In Aspergillus nidulans, SepH, the homolog of CDC7, has been identified as a central component for the initiation of septation prior to actin ring formation. SepH is the kinase upstream of SepL, which is the homolog of SmKIN3, followed by SidB (homologue of DBF2) (36, 37). SIN and its downstream effectors are involved in forming the CR, which is responsible for initiating the formation of septa. To specify the location for septum formation, axial landmark proteins, such as Bud3 and Bud4, are needed, and they recruit septin AspB to the CR (18). Downregulation of *sepH* abolishes septation, whereas hyper‐activation results in the formation of multiple septa (36). This indicates that SepH acts as a positive regulator of SIN, which triggers cytokinesis in *A. nidulans* (38). Comparable phenotypes were observed in *S. macrospora*, where the loss of *Smkin3* or mutation in the ATP-binding site results in the reduction of septa (14). Phosphorylation mutants of SmKIN3, as reported here, show a hyper-septation phenotype that was similarly observed in A. nidulans mutants with hyper‐activation of SepH. Our results are consistent with findings for A. nidulans and N. crassa, which showed that homologs of CDC7 and SmKIN3 act in the same pathway as positive regulators of SIN.

### The phosphorylation state of SmKIN3 affects its localization.

Here, we demonstrate that SmKIN3’s septal localization is altered in mutated phosphorylation strains and STRIPAK deletion mutants, indicating that this process is also mediated by STRIPAK. Furthermore, the STRIPAK-dependent phosphorylation state of SmKIN3 affects its affinity for septal proteins and thus its localization. These findings are consistent with results obtained for fission yeast. There, it was shown that SIN and STRIPAK affect each other by phosphorylation and dephosphorylation (33). SIN phosphorylates guanine exchange factors (GEFs) and GTPases, which in turn phosphorylate the formin Cdc12p. The phosphorylation of Cdc12p is important for the assembly of actin to form the CR. The NDR kinase Sid2p is necessary to phosphorylate the septin Cdc12p and other proteins to form the CR (39). Such phosphorylation at the CR must be tightly regulated to form the final septum.

**FIG 6:**
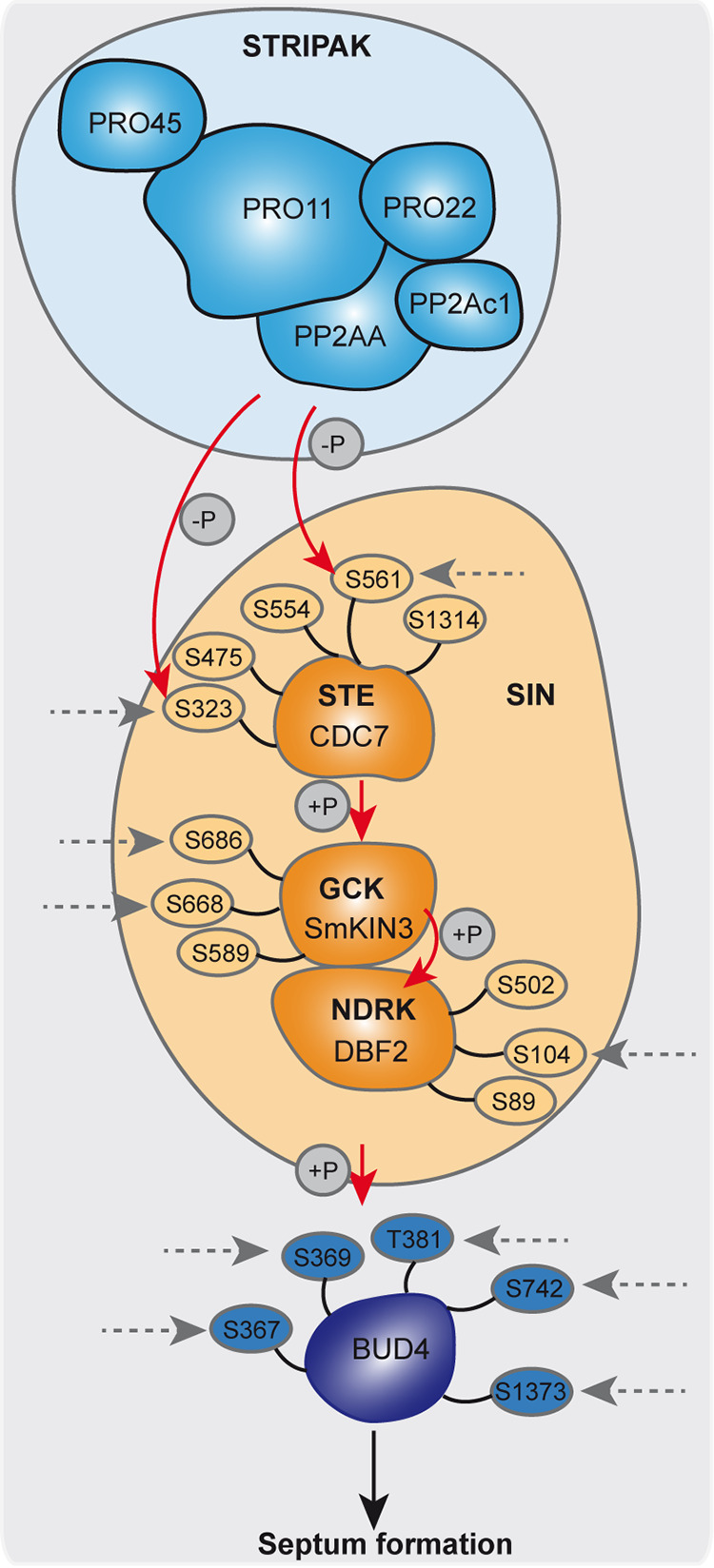
Mechanistic model of STRIPAK-dependent phosphorylation of SmKIN3, as part of the SIN complex. Ovals indicate relevant phosphorylation sites in SIN components and the related BUD4 protein. STRIPAK subunits are light blue, SIN components are orange, and BUD4 is dark blue. STRIPAK-dependent phosphorylation is indicated by gray dashed arrows. Red arrows show direct phosphorylation. This model is based on data from this study and from a recent publication (8).

To summarize our findings, we have designed a schematic mechanistic model, depicted in Fig. 6. The SIN complex acts downstream of STRIPAK, which dephosphorylates CDC7 directly (33). However, STRIPAK acts indirectly on the phosphorylation sites S668 and S686 of SmKIN3, while the phosphorylation of S589 is likely regulated by an unknown phosphatase and kinase. Furthermore, STRIPAK also indirectly phosphorylates S104 in DBF2, while S89 and S502 are not STRIPAK dependent. Finally, from our PRM analysis, we propose that the phosphorylation of all sites from BUD4 depends on STRIPAK signaling.

We hypothesize further that deletion of STRIPAK subunits results in higher phosphorylation of CDC7, which prevents assembly of SIN (33). This results in a lower level of phosphorylated SmKIN3, which consequently is unable to phosphorylate the downstream kinase DBF2. Subsequently, signaling of SIN is impaired, affecting the localization of a RHO4/BUD3 GTPase module assembled by its anillin-type scaffold, BUD4. This localization is critical to form the CR, leading to lower septation (31, 34). Indeed, our quantitative phosphorylation data provide evidence that four phosphorylation sites of BUD4 and the phosphorylation site S104 of DBF2 are dephosphorylated in STRIPAK deletion mutants compared to in the wild type. The phosphorylation site S502 of DBF2, which is the homolog to the phosphorylation site S499 in N. crassa, is not regulated by STRIPAK (Data Set S2). This is consistent with results obtained with A. nidulans. There, localization of septin AspB, which is the homolog of CDC12 in *S. macrospora*, is dependent on the formin SepA (SMAC_04496), the SIN kinase SepH (CDC7), and on its own phosphorylation state.

Dephosphorylation of the conserved threonine at position 68 in SepH was shown to be critical for the timing of septation and localization (40, 41). In N. crassa, phosphorylation analysis revealed that the phosphorylation of DBF-2 is stimulated by SID-1. Interestingly, phospho-deficient and phospho-mimetic mutants of DBF-2 phosphorylation site S499 in N. crassa are nonfunctional *in vivo* and reduce the kinase activity of DBF-2 *in vitro* (16). However, our data indicate that S499 (S502 in *S. macrospora*) seems not to be regulated by STRIPAK but is essential for septum formation in N. crassa (16).

The interaction between components of the SIN cascade is decreased when their dephosphorylation is diminished due to a nonfunctional STRIPAK complex. Moreover, the lack of SIN phosphorylation prevents the recruitment of SIN components to septal pore proteins at the hyphal tip (42). Thus, the interaction of SmKIN3 with SIN and STRIPAK has to be strongly regulated to form septa in a wild-type-like manner, as suggested by our localization experiments with wild-type and mutant strains.

SmKIN3 homologs are conserved from yeasts to humans, and SIN-like pathways have been mentioned in this discussion. We propose that the mechanism of SmKIN3 function described in this study may be applicable to that of homologous networks in other organisms.

## MATERIALS AND METHODS

### Strains and growth conditions.

Electro-competent Escherichia coli cells XL1 Blue MRF′ or chemical-competent E. coli NEB 5-alpha (43, 44) was used for cloning and propagation of recombinant plasmids (45) under standard laboratory conditions (46). All *S. macrospora* strains used in this study are listed in Table S2 in the supplemental material and were grown under standard conditions (47, 48). For further characterization, wild-type and mutant strains were grown at 6°C, 16°C, 27°C, and 30°C under light and dark conditions on *Sordaria* Westergaard’s (SWG) media (49). In all cases, we investigated three technical replicates. For analysis of distribution, SmKIN3 strains were grown for 24 h on solid biomalt-cornmeal medium (BMM)-coated glass. Isogenic and homokaryotic strains were generated by genetic crossing and ascospore isolation (47). To obtain phospho-mutants, the mutated plasmids (Table S1) were transformed into a Δ*Smkin3* strain. Phospho-mutations in the generated strains were verified by PCR analysis and DNA sequencing (Eurofins Genomics, Ebersberg, Germany). The number of ectopically integrated recombinant DNA fragments was verified by Southern hybridization analysis (Fig. S2B).

10.1128/mBio.00658-21.2 TABLE S2 Strains used in this work. Download Table S2, DOCX file, 0.02 MB. Copyright © 2021 Stein et al. 2021 Stein et al. https://creativecommons.org/licenses/by/4.0/ This content is distributed under the terms of the Creative Commons Attribution 4.0 International license.

### *In vitro* recombinant techniques.

Plasmids used in this study are listed in Table S1. pIG1783-*Smkin3-gfp* was created by restricting the plasmid pIG1783 with *Nco*I. *Smkin3* was amplified via PCR with overhangs at the 5′ and 3′ ends containing recognition sites for *Nco*I. Ligation was performed with T4 DNA ligase. For phospho-mimetic and phospho-deficient strains, plasmid pIG1783-*Smkin3-gfp* was used for Q5 mutagenesis (New England Biolabs). With specific primers (Table S3), we generated eight plasmids containing phospho-mimetic and phospho-deficient mutations (Table S1).

10.1128/mBio.00658-21.3 TABLE S3 Oligonucleotides used in this work. Download Table S3, DOCX file, 0.01 MB. Copyright © 2021 Stein et al. 2021 Stein et al. https://creativecommons.org/licenses/by/4.0/ This content is distributed under the terms of the Creative Commons Attribution 4.0 International license.

### Microscopic investigations.

Microscopic experiments were performed using an Axio Imager microscope (Zeiss, Thornwood, NY) coupled with a CoolSNAP HQ camera (Roper Scientific) and a Spectra-X LED lamp (Lumencor) at room temperature. Images were acquired and edited with MetaMorph (version 7.7.0.0; Universal Imaging). Strains were grown on glass slides covered with solid BMM and incubated for 24 to 48 h. GFP fluorescence was analyzed using filter set 49002 (Chroma Technology Corp.; GFP, excitation filter HQ470/40, emission filter HQ525/50, beam splitter T495LPXR). Septa in vegetative hyphae and ascogonial coils were stained using calcofluor white M2R (CFW; Sigma, St. Louis, MO) with a concentration of 1 μg/ml CFW stock solution diluted 1:400 in distilled sterile water. CFW fluorescence was analyzed using Chroma filter set 31000v2 (excitation filter D350/50, emission filter D460/50, beam splitter 400dclp; Chroma Technology Corp., Bellows Falls, VT).

To analyze the distribution of SmKIN3 on septa, at least 50 hyphal tips of the growing front were observed in 10 to 15 independent samples per strain in three different independent strains. Septa were stained with CFW, and SmKIN3-GFP localization was considered only when detected with an exposure time between 100 and 1,000 ms. For analysis of hyper-septation, e.g., double, triple, quadruple, or quintuple septa, we investigated at least 200 single hyphal branches for each strain in three technical repeats. In the case of recombinant strains, three different single ascospore isolates were investigated to exclude side effects from random integration of recombinant DNA. In these cases, at least 200 hyphal branches were counted for each recombinant strain. For the Δ*pro11*, Δ*pp2Ac1*, and Δ*pro22* STRIPAK deletion mutants, we investigated at least 100 hyphal branches from three technical replicates. We counted all septa within 20 μm behind hyphal branches. Septa within a distance of maximum 12 μm were designated hyper-septation septa. Septal distances in hyphae were measured using MetaMorph (version 7.7.0.0; Universal Imaging).

### Peptide selection for targeted quantification of phosphorylation sites.

Based on the results of the global iTRAQ-based (phospho-)proteomic analyses previously performed (8, 9), as well as unpublished results, phosphorylated peptides that showed differential regulation in STRIPAK mutant strains compared to those in the wild type were selected and identified as putative dephosphorylation targets of the STRIPAK complex. In addition, the corresponding nonphosphorylated peptides were selected for quantification, thus enabling calculation of a site occupancy value for the respective phosphorylation sites. As controls, phosphopeptides representing cell division control protein 48 (CDC48; SMAC_00109) and heat shock protein 90 (HSP90; SMAC_04445) were included (Data Set S2), since they showed no regulation in the global experiments and have so far not been described to be functionally connected to the STRIPAK complex.

### Synthesis, purification, and quantification of SIS peptides.

Synthesis of all SIS peptides was performed in-house using a Syro I synthesis unit (MultiSynTech, Witten, Germany) and Fmoc chemistry. Synthesis and subsequent purification were performed as described previously (50). Heavily labeled lysine (^13^C_6_^15^N_2_) and arginine (^13^C_6_^15^N_4_) were incorporated at the C-terminus, and amino acid analysis was applied to determine peptide concentrations (51). A five-point calibration curve of derivatized amino acids, ranging from 5 to 25 pmol/μl, was used for quantification.

Protein extraction, digestion, and normalization and phosphopeptide enrichment were performed as recently reported (8).

### Nano-LC-tandem MS (MS/MS) for PRM of unphosphorylated peptides.

Samples were analyzed on an Ultimate 3000 rapid-separation liquid chromatography nano (RSLCnano) high-performance LC (HPLC) system coupled with a Q Exactive HF mass spectrometer (MS; both from Thermo Scientific). The HPLC was equipped with a trapping column (100 μm by 2 cm, C_18_, PepMap RSLC; Thermo Scientific) for preconcentration and an analytical column (75 μm by 50 cm C_18_, PepMap RSLC; Thermo Scientific) for separation of the peptides. Preconcentration was performed for 5 min at a flow rate of 20 μl/min using 0.1% trifluoroacetic acid (TFA), and separation was performed at a flow rate of 250 nl/min. An optimized binary gradient of solvent A (0.1% formic acid [FA]) and solvent B (84% acetonitrile, 0.1% FA) was used with the following steps: 0 min, 2% B; 5 min, 2% B; 10 min, 5% B; 50 min, 9% B; 73 min, 15% B; 100 min, 21% B; and 115 min, 45% B. This was followed by two washing steps for 5 min at 95% B and 20 min of equilibration at 2% B. The MS was operated in PRM mode at a resolution of 60,000 (at 200 *m/z*) with a fixed first mass of 150 *m/z*. The AGC target was set to 1 × 10^6^ and a maximum injection time of 118 ms. Targeted precursors were isolated with a quadrupole isolation width of 0.4 *m/z* and fragmented with a normalized collision energy of 27. PRM acquisition was scheduled with a retention time window of 2 min per target.

### Nano-LC-MS/MS for PRM of phosphorylated peptides.

Enriched phosphorylated peptides were analyzed with the same instrumentation and settings as described above. The LC gradient was optimized to suit the targeted phosphopeptides, and the steps were modified as follows: 0 min, 3% B; 5 min, 3% B; 15 min, 7% B; 37 min, 10% B; 90 min, 20% B; 110 min, 27% B; and 120 min, 45% B. This was followed by the same washing and equilibration steps as above.

### PRM development and analysis.

To verify the linearity of response and determine the limit of blank (LOB), LOD, and LLOQ, response curves of both phosphorylated and nonphosphorylated SIS peptides were acquired. In both cases, a background matrix was generated by pooling aliquots of all individual samples. SIS peptides were spiked in at eight different concentrations, covering a range of 3 orders of magnitude. Based on prior determination of individual SIS response factors, the highest concentrations for nonphosphorylated SIS peptides varied between 54 fmol and 2.73 pmol on column, while the lowest concentrations were between 13 and 667 amol on column. For the analysis of the individual samples, a total amount of nonphosphorylated SIS peptides ranging from 0.05 fmol to 60 fmol was spiked into 3 μg of the total protein digest, depending on the expected endogenous concentration (all concentrations are given in Data Set S1).

For phosphorylated SIS peptide calibration curve measurements, a total of eight dilution points were generated with SIS peptide concentrations ranging from 600 amol to 2.4 pmol in 200 μg of the background matrix for the best responding peptides, as detailed in Data Set S1.

In addition, six phoshphoisomeric stable isotope-labeled (SIL) peptides were included in the assay, but no endogenous peptide was detected. These peptides could be further used to rule out the presence of these isomers through knowledge of their retention time and diagnostic transitions. The median CV of the biological replicates was calculated as 16.3% for all nonphosphorylated and as 16.1% for all phosphorylated peptides. The lowest average site occupancy was detected at 0.06% on the phosphorylation sites S369 in the HSP90 protein SMAC_04445. This site was included as a negative control, and its occupancy showed no significant difference between the wild type and any of the deletion strains. The phosphorylation site S2349 in the phosphatidylinositol 3-kinase TOR2 (SMAC_03322) exhibited the highest site occupancy, with 92.2% measured in the wild type.

All calibration curve samples were subjected to TiO_2_-based phosphopeptide enrichment and nano-LC-MS/MS measurement in technical triplicates (16.7% of eluate per replicate) as detailed above. Additionally, two replicates without the SIS spike-in (DS0) were processed in parallel to appropriately determine background signal levels. For the final analysis of individual samples, a total amount of 410 amol to 4.1 fmol was spiked into 450 μg of the protein digest, followed by phosphopeptide enrichment and nano-LC-MS/MS measurement of 25% of the eluates after enrichment.

Skyline software (version 4.1 [52]) was used to analyze all of the PRM data. The top three most suitable transitions of every light and SIS peptide pair were chosen. All data were manually inspected for correct peak detection, retention time, and integration, and peak areas were exported. R software (53) (version 3.5.3) was used for data analysis and calibration curve measurements, and LOB and LOD were calculated using the MSstats package (54). The light/heavy (L/H) peak area ratios were used to determine the concentration (*c*) of phosphorylated and nonphosphorylated peptide per sample. The phosphorylation site occupancy was calculated using the following formula:

$$\frac{c(\text{phosphorylated peptide})}{c\left(\text{phosphorylated peptide}\right)+c(\text{nonphosphorylated peptide})}\times 100=\text{site occupancy }\left(\%\right)$$

Statistical comparison of the phosphorylation site occupancy between the wild-type and knockout strains was performed using a two-sided Student *t*-test.

### Data availability.

All targeted proteomics data and raw files are available through the Panorama repository (55) under the data set identifier PXD023130 and via https://panoramaweb.org/SmKIN3.url.
