# Supplementary figures and images for: Targeted Quantification of Phosphorylation Sites Identifies STRIPAK-Dependent Phosphorylation of the Hippo Pathway-Related Kinase SmKIN3
Source: mBio. 2021 May 4;12(3):e00658-21. doi: 10.1128/mBio.00658-21 (PMC8262875; doi:10.1128/mBio.00658-21)

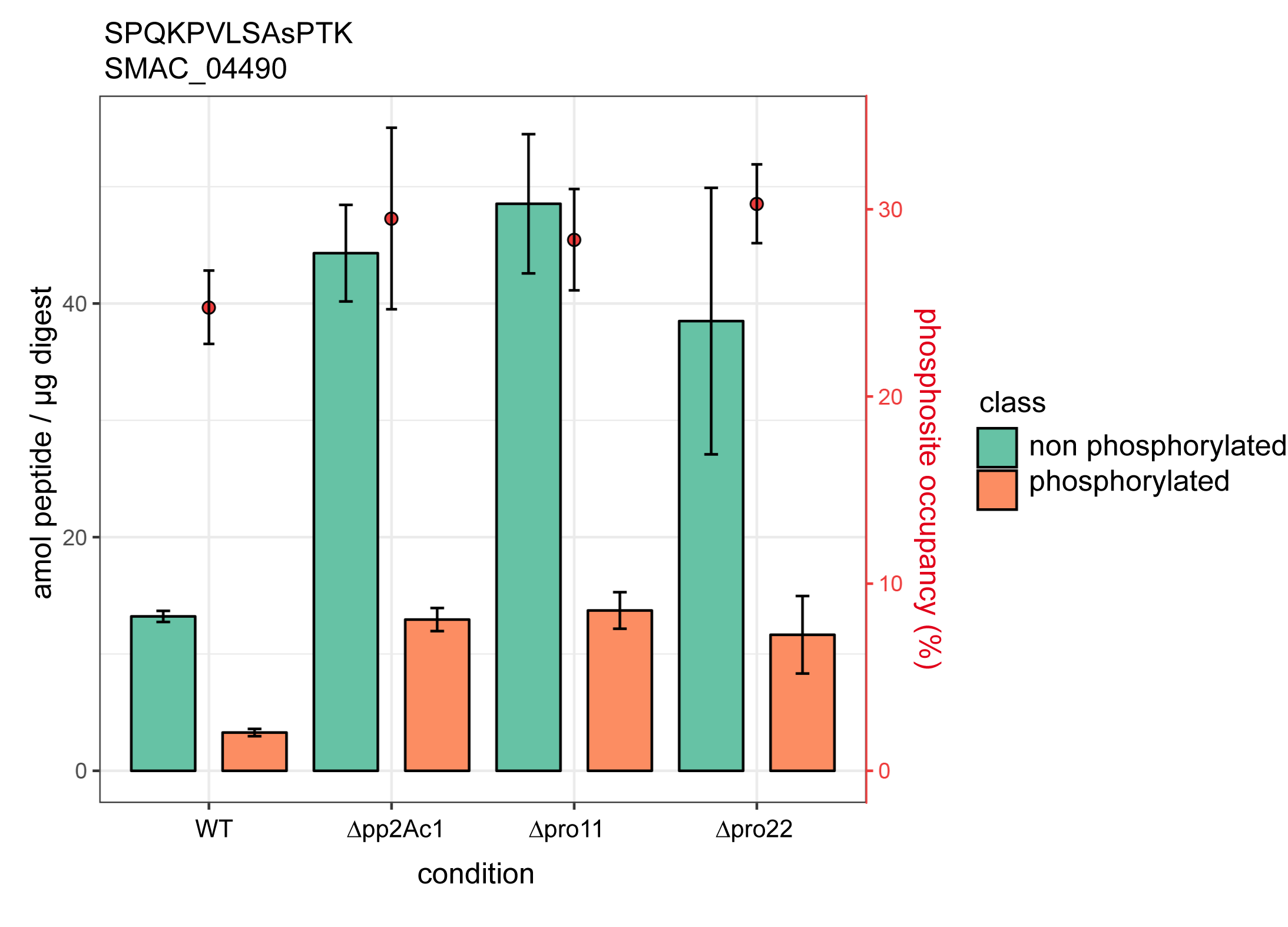

Supplement: FIG S1 [file mbio.00658-21-sf001.tif]

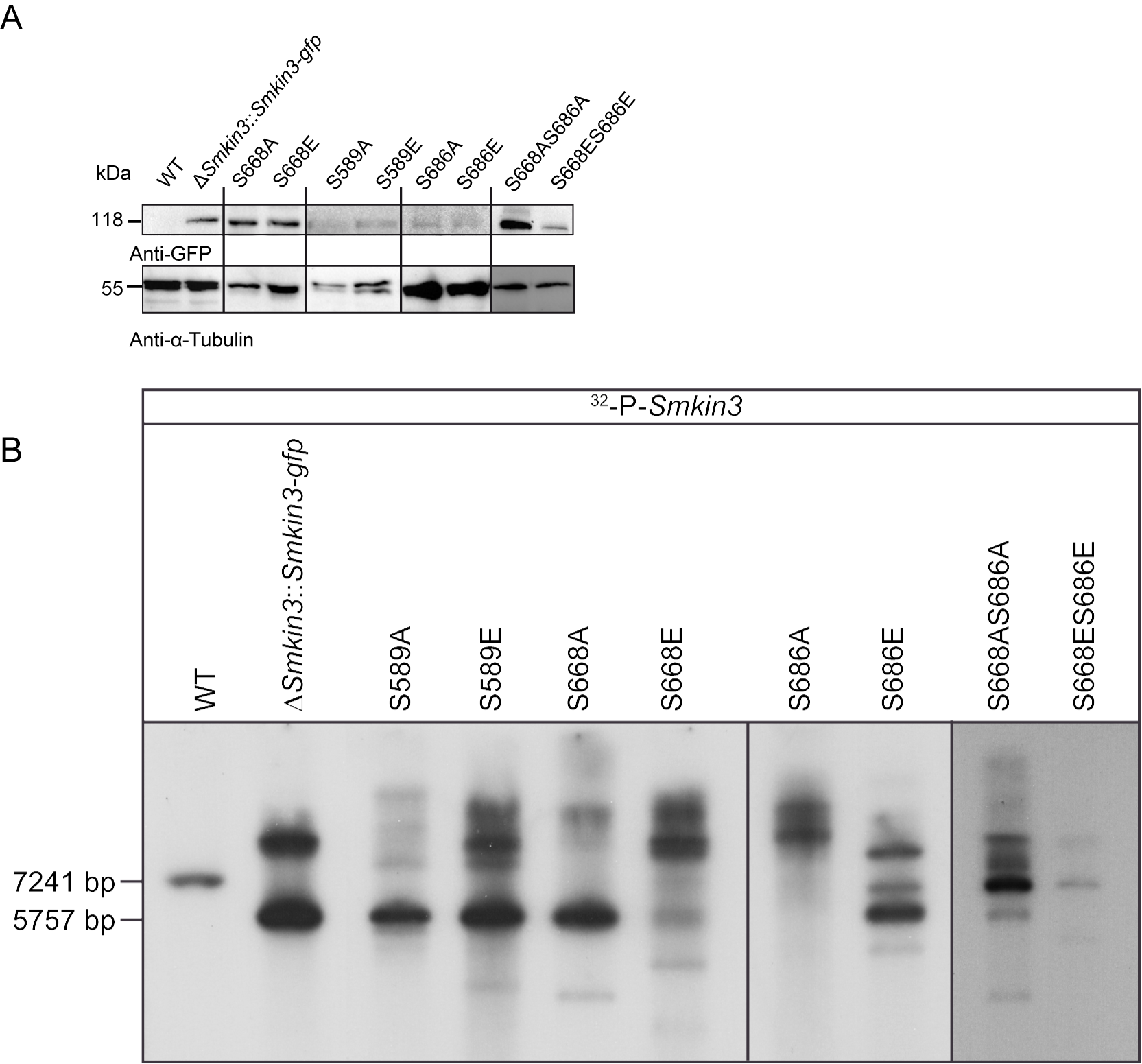

Supplement: FIG S2 [file mbio.00658-21-sf002.tif]

**A**

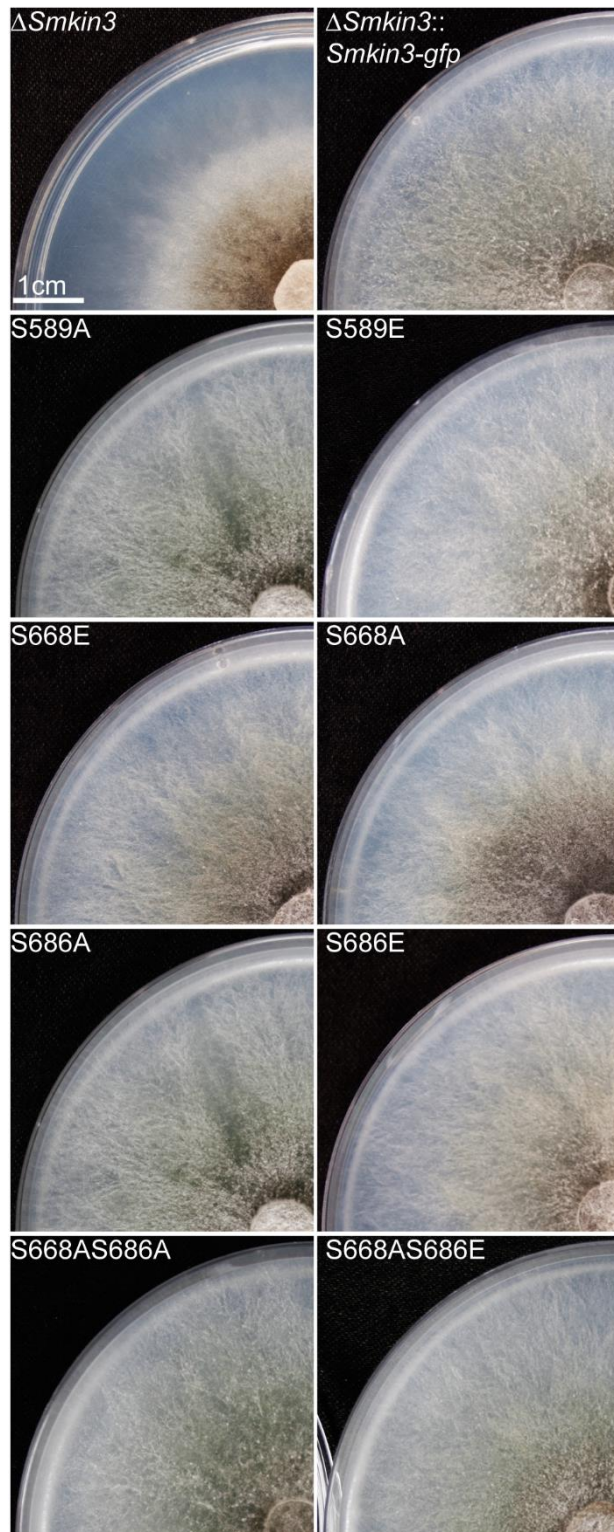

**B**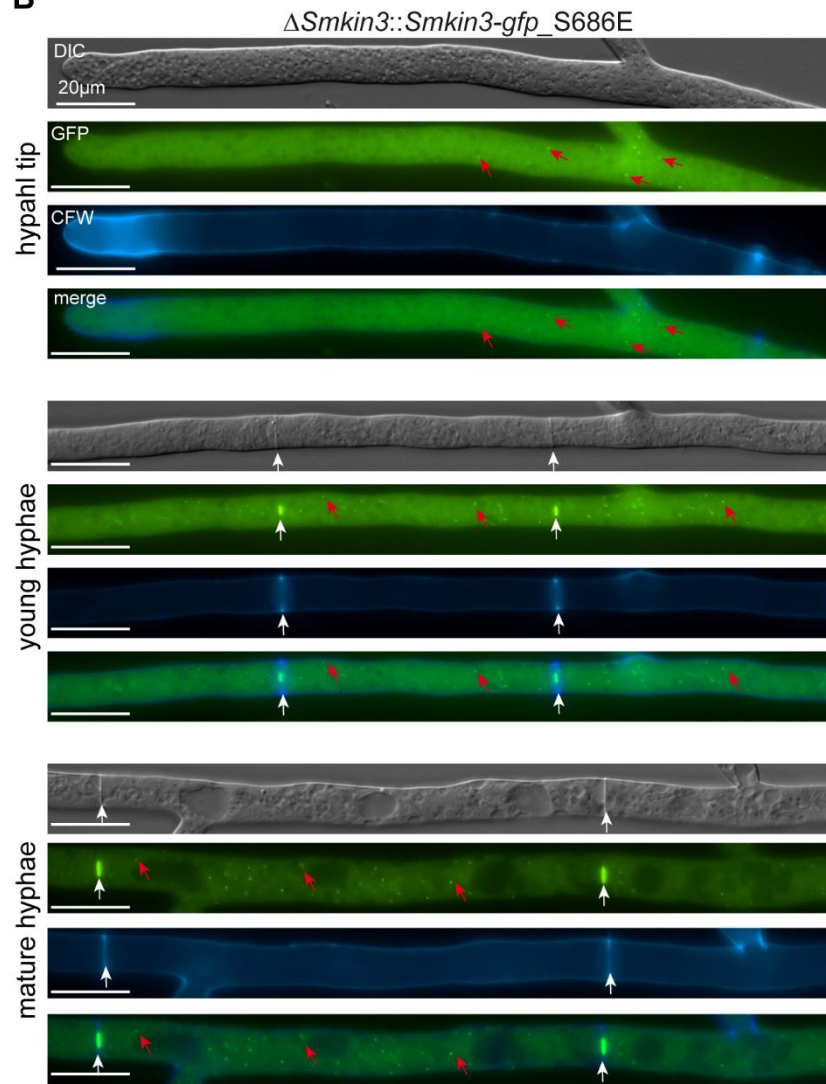

Supplement: FIG S3 [file mbio.00658-21-sf003.pdf]

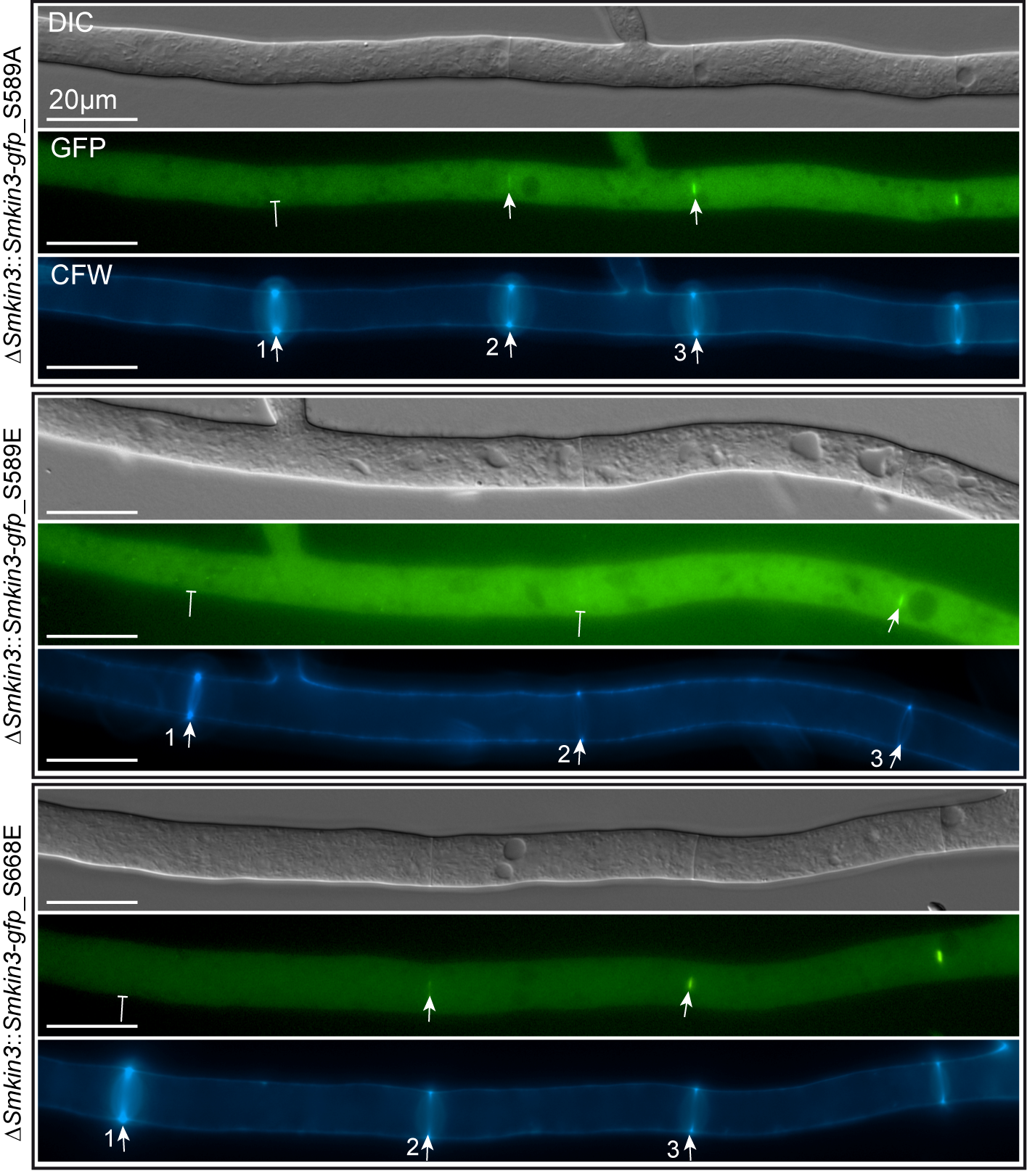

Supplement: FIG S4 [file mbio.00658-21-sf004.tif]
