# Supplementary material for: Targeted Quantification of Phosphorylation Sites Identifies STRIPAK-Dependent Phosphorylation of the Hippo Pathway-Related Kinase SmKIN3
Source: mBio. 2021 May 4;12(3):e00658-21. doi: 10.1128/mBio.00658-21 (PMC8262875; doi:10.1128/mBio.00658-21)
Supplement: TABLE S1 [file mbio.00658-21-st001.docx]

**Table S1** Plasmids used in this work

| **Plasmid** | **Characteristics** | **Reference** |
| --- | --- | --- |
| pIG1783nat | gpd(p)::gfp::trpC(t)::nat^r^ | Teichert, unpublished |
| pIG1783-*Smkin3-gfp* | gpd(p)::Smkin3::gfp::trpC(t)::nat^r^ | This work |
| pVS118-15 | gpd(p)::Smkin3S668E::gfp::trpC(t)::nat^r^ | This work |
| pVS121-4 | gpd(p)::Smkin3S668A::gfp::trpC(t)::nat^r^ | This work |
| pVS122-2 | gpd(p)::Smkin3S589A::gfp::trpC(t)::nat^r^ | This work |
| pVS123-16 | gpd(p)::Smkin3S589E::gfp::trpC(t)::nat^r^ | This work |
| pVSo1-6 | gpd(p)::Smkin3S686A::gfp::trpC(t)::nat^r^ | This work |
| pVSo1-15 | gpd(p)::Smkin3S686E::gfp::trpC(t)::nat^r^ | This work |
| pVS124-1 | gpd(p)::Smkin3S668AS686A::gfp::trpC(t)::nat^r^ | This work |
| pVS125-25 | gpd(p)::Smkin3S668ES686E::gfp::trpC(t)::nat^r^ | This work |
