# Supplementary material for: Targeted Quantification of Phosphorylation Sites Identifies STRIPAK-Dependent Phosphorylation of the Hippo Pathway-Related Kinase SmKIN3
Source: mBio. 2021 May 4;12(3):e00658-21. doi: 10.1128/mBio.00658-21 (PMC8262875; doi:10.1128/mBio.00658-21)
Supplement: TABLE S2 [file mbio.00658-21-st002.docx]

**Table S2** Strains used in this work

| **Strain** | **Relevant genotype** | **Relevant phenotype** | **Reference** |
| --- | --- | --- | --- |
| R19027 | Wild type | fertile | * culture collection |
| S70823 | *fus* | fertile, brown ascospores | * culture collection |
| ∆pro11/fus | *∆pro11::hyg^r^/fus* | sterile | * culture collection |
| A1844 | Δ*pp2Ac1::hph^r^* | sterile | (Beier et al. 2016) (mBio 7: 00870-00816, 2016, doi: 10.1128/mBio.00870-16) |
| S123556 | Δ*pp2Ac1::hph^r^, fus* | sterile | * culture collection |
| D2273 | *∆Smkin3::hyg^r^* | sterile | (Radchenko et al. 2018) (Genetics 210: 137-153, 2018, https://doi.org/10.1534/genetics.118.301261) |
| TVS9DESI11 | *∆Smkin3::hyg^r^, gpd(p)::Smkin3::gfp::trpC(t)::nat* | fertile | This work |
| S672 | *Wt::gpd(p)::Smkin3::gfp::trpC(t)::nat^r^* | fertile | This work |
| ∆pro11S1 | *∆pro11:: hyg, gpd(p)::Smkin3::gfp::trpC(t)::nat^r^* | sterile | This work |
| ∆pp2Ac1S1 | *∆pp2Ac1:: hyg^r^, gpd(p)::Smkin3::gfp::trpC(t)::nat* | sterile | This work |
| TVS87A4S3 | *∆Smkin3:: hyg^r^::gpd(p)::Smkin3S668E::gfp::trpC(t)::nat* | fertile | This work |
| TVS87CFS4 | *∆Smkin3:: hyg^r^::gpd(p)::Smkin3S668E::gfp::trpC(t)::nat* | fertile | This work |
| TVS87B1S2 | *∆Smkin3:: hyg^r^::gpd(p)::Smkin3S668E::gfp::trpC(t)::nat* | fertile | This work |
| TVS85D2S2 | *∆Smkin3:: hyg^r^::gpd(p)::Smkin3S668A::gfp::trpC(t)::nat* | fertile | This work |
| TVS85A1S3 | *∆Smkin3:: hyg^r^::gpd(p)::Smkin3S668A::gfp::trpC(t)::nat* | fertile | This work |
| TVS89B2S2 | *∆Smkin3:: hyg^r^::gpd(p)::Smkin3S668A::gfp::trpC(t)::nat* | fertile | This work |
| S1983 | *∆Smkin3:: hyg^r^::gpd(p)::Smkin3S589A::gfp::trpC(t)::nat* | fertile | This work |
| TVS98AS5 | *∆Smkin3:: hyg^r^::gpd(p)::Smkin3S589A::gfp::trpC(t)::nat* | fertile | This work |
| TVS98BS20 | *∆Smkin3:: hyg^r^::gpd(p)::Smkin3S589A::gfp::trpC(t)::nat* | fertile | This work |
| TVS88C4S2 | *∆Smkin3:: hyg^r^::gpd(p)::Smkin3S589E::gfp::trpC(t)::nat* | fertile | This work |
| TVS88D2S2 | *∆Smkin3:: hyg^r^::gpd(p)::Smkin3S589E::gfp::trpC(t)::nat* | fertile | This work |
| TVS88CS10 | *∆Smkin3:: hyg^r^::gpd(p)::Smkin3S589E::gfp::trpC(t)::nat* | fertile | This work |
| TVSO1 B2-3 S8 | *∆Smkin3:: hyg^r^::gpd(p)::Smkin3S686A::gfp::trpC(t)::nat* | fertile | This work |
| TVSo1 C1(1) s 18 | *∆Smkin3:: hyg^r^::gpd(p)::Smkin3S686A::gfp::trpC(t)::nat* | fertile | This work |
| TVSo1 D2(1) S2 | *∆Smkin3:: hyg^r^::gpd(p)::Smkin3S686A::gfp::trpC(t)::nat* | fertile | This work |
| TVSo2 D1-2(1) S8 | *∆Smkin3:: hyg^r^::gpd(p)::Smkin3S686E::gfp::trpC(t)::nat* | fertile | This work |
| TVSo2 B4 | *∆Smkin3:: hyg^r^::gpd(p)::Smkin3S686E::gfp::trpC(t)::nat* | fertile | This work |
| TVSo2 H2 S11 | *∆Smkin3:: hyg^r^::gpd(p)::Smkin3S686E::gfp::trpC(t)::nat* | fertile | This work |
| S5060 | *∆Smkin3:: hyg^r^::gpd(p)::Smkin3S668AS686A::gfp::trpC(t)::nat* | fertile | This work |
| TVS99.2CF | *∆Smkin3:: hyg^r^::gpd(p)::Smkin3S668AS686A::gfp::trpC(t)::nat* | fertile | This work |
| TVS99.2AF | *∆Smkin3:: hyg^r^::gpd(p)::Smkin3S668AS686A::gfp::trpC(t)::nat* | fertile | This work |
| S6008 | *∆Smkin3:: hyg^r^::gpd(p)::Smkin3S668ES686E::gfp::trpC(t)::nat* | fertile | This work |
| TVS100.2DF | *∆Smkin3:: hyg^r^::gpd(p)::Smkin3S668ES686E::gfp::trpC(t)::nat* | fertile | This work |
| TVS100.2BFP | *∆Smkin3:: hyg^r^::gpd(p)::Smkin3S668ES686E::gfp::trpC(t)::nat* | fertile | This work |

*Culture collection of the department for General and Molecular Botany, Ruhr-University Bochum
