# Supplementary material for: Targeted Quantification of Phosphorylation Sites Identifies STRIPAK-Dependent Phosphorylation of the Hippo Pathway-Related Kinase SmKIN3
Source: mBio. 2021 May 4;12(3):e00658-21. doi: 10.1128/mBio.00658-21 (PMC8262875; doi:10.1128/mBio.00658-21)
Supplement: TABLE S3 [file mbio.00658-21-st003.docx]

**Table S3** Oligonucleotides used in this work

| **Oligonucleotide** | **Sequence (5’ – 3’)** | **Specificity** |
| --- | --- | --- |
| Hph1MN | cgatggctgtgtagaagtactcgc | *hph* |
| Hph2MN | atccgcctggacgactaaaccaa | *hph* |
| 1751 | gccatattttcctgctctcc | *Pgpd* forward |
| 1757 | agctgacatcgacaccaacg | *TtrpC* reverse |
| *Smkin3*-fw-VS | agatccatcgcgacatcaaagc | *SmKin3* |
| *Smkin3*-rv-VS | tggtaagctccttcaggatc | *SmKin3* |
| *Smkin3*-NcoI-fw | ccatggccgacgaaggagtcgccaacc | *SmKin3* |
| *Smkin3*-NcoI-rv | ccatggCagatccggcaacagccccccaccga | *SmKin3* |
| Pro11-21 | AAGCGCGCTTGCCAGTCGCTGC | 5´flank of *pro11* |
| Nr. 69, AB | TCTAGAGCGCCGAATCAAACCAAT | 5´flank of *pp2Ac1* |
| Q5-kin3-S668A-fw | ACAGCAGCACgccGCCCCGTCTC | *Smkin3* S668 codon |
| Q5-kin3-S668A-rv | AAGAGCTGGGGCTCCTTCTTTTGGG | *Smkin3* S668 codon |
| Q5-kin3-S668E-fw | ACAGCAGCACgagGCCCCGTCTC | *Smkin3* S668 codon |
| Q5-kin3-S668E-rv | AAGAGCTGGGGCTCCTTC | *Smkin3* S668 codon |
| Q5-kin3-S589A-fw | TCTTTCCGCCgccCCGACAAAACTGATGAGC | *Smkin3* S589 codon |
| Q5-kin3-S589AE-rv | ACCGGCTTTTGCGGGGAG | *Smkin3* S589 codon |
| Q5-kin3-S589E-fw | TCTTTCCGCCgagCCGACAAAACTGATGAGCC | *Smkin3* S589 codon |
| Q5-S686A_fw | GCAGCAACTCgCCCACCCGGT | *Smkin3* S686 codon |
| Q5-S686A_rv | TGGAGGGGTGGCGGGTTTT | *Smkin3* S686 codon |
| Q5-S686E_fw | GCAGCAACTCgagCACCCGGTCCTCCAG | *Smkin3* S686 codon |
| Q5-S686E_rv | TGGAGGGGTGGCGGGTTT | *Smkin3* S686 codon |
